# Free-living amoebae: a journey into historical aspects and to current discoveries

**DOI:** 10.1590/0074-02760240246

**Published:** 2025-02-24

**Authors:** Helena Lúcia Carneiro Santos

**Affiliations:** 1 Fundação Oswaldo Cruz-Fiocruz, Instituto Oswaldo Cruz, Laboratório de Doenças Parasitárias, Rio de Janeiro, RJ, Brasil

**Keywords:** freshwater amoebae, *Acanthamoeba* sp., host-parasite interaction, taxonomy

## Abstract

Free-living amoebas (FLA) are ubiquitous protists found in the environment. They have shown exceptional resistance to environmental challenges and play significant roles in controlling microbial populations through their predatory behaviour and microbicidal activity in both soil and aquatic ecosystems environments. However, although rare, a limited group of FLA can cause serious infections in the central nervous system and other diseases, particularly in immunocompromised individuals with high mortality rates. They can also cause keratitis in otherwise healthy individuals. This review offers a comprehensive overview of freshwater naked amoebae but does not cover all aspects in detail. Its goal is to provide a historical context for our current understanding while addressing the most critical elements of FLA biology, their pathogenic potential, and their interactions with important human pathogens.

Amoebae are a group of protists that exhibit irregular shapes by extending pseudopods (cytoplasm expansion), supported by their cytoskeleton, composed of actin-myosin or tubulin elements. These pseudopods are used for movement or capture food.^1^ ^,^ ^2^ Amoebae can be found across all branches of the eukaryotic trees,^3^ ^,^ ^4^ ^,^ ^5^ as some amoeboid organisms have genetic relationships closer to plants or metazoans.^6^ Free-living amoebae (FLAs) are non-monophyletic, single-celled eukaryotic commonly found in natural and artificial aquatic environments, as well as in moist soil and within the microfauna of vertebrates and invertebrates.^7^ ^,^ ^8^ ^,^ ^9^ ^,^ ^10^ ^,^ ^11^ Notably, some FLAs can enter a temporary multicellular phase when food is scarce.^12^ They are extremely diverse and widely distributed across all continents, including the harsh conditions of Antarctica.^9^ ^,^ ^13^ ^,^ ^14^ ^,^ ^15^ Despite being studied since the advent of microscopy, our understanding of these organisms remains limited.

Revisiting the history of free-living amoeba and reflecting on Aragão’s research and hierarchical classification

The identification and classification of FLAs play a crucial role in understanding their diversity, evolutionary relationships, and ecological significance.^9^ Research on FLAs has a significant historical legacy that dates to the mid-eighteenth century. In 1755, the German naturalist August Johann Rosel von Rosenhof (1705-1759) provided detailed illustrations and descriptions of a freshwater amoeba through a microscope, naming it “small proteus”, which resembled *Amoeba proteus* according to his illustrations.^16^ Later, the Swedish naturalist Carl Linnaeus (1707-1778) classified it as *Chaos proteus*.^17^ In 1838, the term “amoeba” was first attributed to this group of organisms by Christian Gottfried Ehrenberg (1785-1876), who established the genus Amoeba in Germany.^18^ Two years later, Felix Dujardin (1801-1860) described “Limax ameba”, in France.^19^ In 1838, Christian Gottfried Ehrenberg (1795-1876), initiated the first attempt to classify amoeboid protists, creating sections based on pseudopod structure (lobopoda, filopodia, reticulopodia and axopoda) and classifying families such as Amoebae and Arcellina.^20^ ^,^ ^21^ ^,^ ^22^ Between 1841 and 1858, a new classification was developed that used the term rhizopods to distinguish amoebae based on the presence or absence of shells, separating freshwater from marine representatives. Subsequently, amoeboid protists were primarily classified based on differences in pseudopod structure. The Rhizopoda class was shared into three orders: Lobosa, Radiolaria, and Reticulosa.^22^ ^,^ ^23^ Over the subsequent decades, the description of new species, classes, orders, and families, often accompanied by multiple synonyms, led to confusion in distinguishing historical amoeboid species. In 1879, Joseph Leidy (1823-1891) proposed grouping all “common” giant freshwater amoebae into a single species, suggesting the name *A. proteus*, marking a period of extensive synonymy.^24^ In 1899, Franz Schardinger (1853-1920) described an amoeba that could transform into a flagellate form, naming it *Amoeba gruberi*.^25^ Later, in 1900, French scientist Pierre Augustin Dangeard (1862-1947) described the mitotic process in amoebae, highlighting the complexity of their classification: “nothing is more difficult and challenging than the classification of amoeba”.^26^ This sentiment was echoed by other researchers,^27^ ^,^ ^28^ including Henrique de Beaurepaire Rohan Aragão (1879-1956), who conducted one of Brazil’s first studies on freshwater amoebae. His research, published in the inaugural edition of the Memórias do Instituto Oswaldo Cruz in 1909, meticulously detailed the biology and a unique form of nuclear division in an amoeba isolate found in freshwater near the Oswaldo Cruz Foundation campus laboratory. Aragão observed two distinct nuclear division processes: the first involved small rod-shaped chromosomes scattered irregularly along the spindle, fusing to form a solid mass of chromatin, representing the daughter plates, while the second involved chromosomes arranged in a definitive equatorial plate. He also described the amoeba’s morphological details, including its trophozoite form, size, pseudopodia, nucleus and vacuoles. This amoeba was named *Amoeba Diplomitica* following microscopic analyses of fresh and stained samples using ferric hematoxylin.^29^ However, it was not recognised as a valid species according to the rules of that time, as the identified characteristics were insufficient to define a species, especially due to the inappropriate cytological staining technique used.^30^ Although not officially recognised, Aragão’s research represents one of many similar studies in protozoology, particularly regarding freshwater amoebae (amphizoic and non-parasitic), paving the way for the work of many scientists in understanding the FLAs. Over the years, research has focused on establishing practical identification rules based on comprehensive analyses of morphological data, ultrastructure, and isoenzyme patterns, particularly, regarding amoeba movement, cytoplasmic flow, nuclear structure, division, and life cycle, which significantly contributed to our current understanding of the morphology of FLAs.^31^ ^-^ ^37^

In the 1930s, there was a decline in research related to in the FLAs. However, some researchers described the phagocytic capacity of freshwater amoebae in bacterial cultures, as well as their selective phagocytosis of red blood cells in cell cultures.^38^ ^,^ ^39^ Cytopathic effects were observed also observed in cell culture, although these were initially considered environmental contaminants.^40^ ^,^ ^41^ Subsequent studies revealed the presence of amoebae in human nose, throat, and lung fluids.^42^ ^,^ ^43^ When these amoebae were introduced into the noses of mice, they infiltrated the mucous membrane, migrated to the brain, and caused fatal meningitis, resulting in the death of all affected animals.^44^ This discovery prompted further research focusing on the detection of potentially pathogenic FLAs, such as *Acanthamoeba* sp. and *Naegleria* sp., which are responsible for primary amoebic meningoencephalitis and granulomatous amoebic meningoencephalitis. The occurrence of human infections by free-living amoebae was demonstrated a few years later.^45^ ^,^ ^46^ ^,^ ^47^ In the 1960s, the first fatal meningitis due to *Naegleria fowleri* was recorded.^46^ Subsequently, brain abscesses were reported,^48^ ^,^ ^49^ ^,^ ^50^ and in 1974, a case of corneal infection caused by *Acanthamoeba* sp. was documented.^51^ Notably, a retrospective study revealed that the first cases of primary amoebic meningoencephalitis occurred in 1937.^52^ *Balamuthia mandrillaris* was identified in the brain of a baboon with meningitis in the United States in 1986^53^ and was officially characterised in 1993.^54^ In addition, there are other species of free-living amoebae associated with human infectious diseases, including *Sappinea pedata*, *Vermamoeba vermiformes* (formerly *Hartmannella vermiformis*), *Vanellas* sp., *Vahlkampfia* sp., and, more recently, *Paravahlkampfia francinae*.^55^ ^,^ ^56^ ^,^ ^57^ ^,^ ^58^ These reports underscore the critical need for ongoing research and surveillance in the field of infectious diseases caused by FLAs.

Amoebic organisms represent a general form of life rather than a specific taxonomic group. Although these organisms may exhibit morphological similarities, such similarities can result from phenotypic convergence or be influenced by the conditions of the culture medium and the source of isolation. Freshwater amoebae were traditionally categorised within the subphylum Sarcodina and superclass Rhizopodea.^59^ However, analyses of small ribosomal subunit RNA (SSU-rRNA) have shown that Sarcodina does not form a monophyletic group.^60^ This finding has prompted a new era in the classification of these protozoans, particularly at the end of the 20th century. The nomenclature of amoebae has been repeatedly, often radically, based on advancements in microscopic techniques, ultrastructure, and DNA analysis.^4^ ^,^ ^5^ Recently, the International Society of Protozoologists proposed a new classification system based on morphological, biochemical, DNA sequencing, and phylogenetic approaches.^5^ The integration of these data has provided new perspectives on the identification of freshwater amoebae and allowed for a more validated classification, leading to revisions and improvements in the classification of FLAs and their relationships among higher-level groups. As a result, FLAs have been classified into four clades (Amoebozoa, Excavata, Rhizaria and Opisthokonta) in the eukaryotic tree of life.^4^ ^,^ ^5^ Many low-level taxa have been established, and the analysis of SSU-rDNA sequences has significantly enhanced our understanding of freshwater amoebae distributions.^60^ ^,^ ^61^ Furthermore, results based on multigene analyses indicate that Naked Lobose Amoeba (Amoebozoa: Lobosa), the most common group in moist soils and freshwater habitats, are robust, particularly regarding the genera and species responsible for opportunist infections in animals.^62^ ^,^ ^63^ ^,^ ^64^ Among these, *Acanthamoeba* spp. *Balamuthia mandrilaris* and *Vannella* sp. are classified within Supergroup Amoebozoa, Class Lobosea (sub-division: Acanthamoebidae and Discosea) and Sappinia (sub-division:Thecamoebidae), while *N. fowleri* and *P. francinae* are classified under Excavata (Heterolobosia:Vahlkampfiidae)^5^ (Table).

**TABLE:** Summary of free-living amoebae taxonomy based on recent revisions^4^ ^,^ ^5^

Eukaryote	Super group	Major groups and genera	Potential pathogenic
	Amoebozoa	Tubuliea (formerly part of Lobosa) *Amoebae*, *Chaos*, *hydramoeba*, *Metachaos Trichamoeba*, *Glaeseri*, *Hartmannella*, *Polychaos*, *Saccamoeba*, *Flabellula*, *Gephyramoeba*, *Leptomyxa*, *Rhizamoeba*, *Trichosphaerium*, *echinamoeba*, *Vermamoeba* Discosea (formerly part of Lobosa) *Acanthamoeba*, *Protoacanthameba*, *Balamuthia*, *Discomoeba*, *Clydonella*, *Hollandella*, *Korotnevella*, *Dactylamoeba*, *Lingulmoeba*, *Mayorella*, *Neoparamoeba*, *Paramoeba*, *Platyamoeba*, *Sappinia*, *Thecamaoeba*, *Unda*, *Vannella*, *Vexilliera* Conosa *Endamoeba*, *Entamoeba*, *Filamoeba*, *iodamoeba*, *Mastigamoebam Martigina*, *Phreatamoeba*, *Mastigella*, *Pelomyxa*, *Dictyostelium*, *physarum*	*Vermamoeba* *vermiformis* *Acantamoeba* spp. *B. mandrilaris* *Sappinia pedata* *Vanella* sp.
	Rhizaria	Cercozoa Filosa: Monadofilosa, Gyromitus, Paulinella, Granofilosea, Chlorarachniophyceae Endomyxa: Proteomyxidea, Order Aconchulinida, Pseudosporida, reticulosida, Gromiidea Foraminifera Radiolaria	-
	Excavates	Discoba Heterolobosea Neovahlkampfiidae: *Neovahlkampfia* Vahlkampfiidae: *Heteramoeba*, *Naegleria*, *Tetramitus*, *Vahlkampfia*, *Paravahlkampfia*, *Neovahlkampfia* Gruberellidae: *Stachyamoeva*, *Bruberella* Acrasidae: *Acrasis*, *Pocheina*, *Allovahlkampfia*	*Vahlkampfia* sp. *Naegleria fowlery* *Paravahlkampfia* *francinae*

In contrast, non-parasitic amoebae species, particularly those found in wet soils and aquatic environments, are seldom studied despite their widespread environmental presence and morphological diversity. Research in this area remains slow and challenging due to a lack of DNA sequences in databases, a shortage of trained taxonomists, limited culture types, and insufficient background information.^63^ FLAs, such as filose and reticulose cercozoans, are represented in the Rhizaria class, with a scattered distribution across different phylogenetic groups. The estimated diversity of FLAs related to Rhizaria and their reliable affiliations remain unresolved.^5^ Some authors have postulated that their diversity may be attributed to prokaryotic lateral gene transfer or that the sampling taxa has been extremely limited.^65^ ^,^ ^66^

It is noteworthy that the diversity of these amoebas is crucial for understanding their biological and ecological roles in the environment. Additionally, in the last decade, new opportunistic free-living amoebae species have been identified as causing diseases in humans and other animals through molecular diagnostics, such as real-time polymerase chain reaction (PCR) and DNA sequencing.^55^ ^,^ ^56^ ^,^ ^58^ ^,^ ^67^

Opportunistic free-living amoebae

Pathogenic and opportunistic free-living amoebae belonging to the genera *Naegleria*, *Balamuthia*, *Acanthamoeba*, *Sappinia*, and *Vahlkampfia* are mitochondria-bearing, aerobic, eukaryotic protists.^9^ ^,^ ^11^ ^,^ ^53^ ^,^ ^57^ ^,^ ^67^ These amoebae can exist as free-living organisms in nature and sometimes parasitise host tissue and are therefore called amphizoic. Infections in humans and other animals are generally not well known or clinically recognised, with the majority of diagnoses made post-mortem, leading to many cases going unrecognised.

*Acanthamoeba* spp. and *B. mandrillaris* can cause an insidious chronic granulomatous disease known as granulomatous amebic encephalitis (GAE) in both immunocompetent and immunosuppressed individuals and animals.

*Balamuthia mandrillaris* leads to a chronic and subacute central nervous system (CNS) disease that develops over a period ranging from two weeks to two years. Many patients with GAE have developed skin lesions on the face, limbs, and hydrocephalus, ulcers in the sinus cavities and rhinitis. Otitis media can occur, and the infection may disseminate to the lungs, kidneys, adrenal glands and uterus.^68^ ^-^ ^72^ However, CNS involvement does not always occur. Since *B. mandrillaris* was identified from the brain tissue of a mandrill baboon that died of meningoencephalitis at the San Diego Zoological, cases have been reported worldwide.^9^ ^,^ ^11^ ^,^ ^54^ In contrast, *N. fowleri* can cause a severe and often fatal disease called primary amoebic meningoencephalitis (PAM), which results in necrotising, hemorrhagic inflammation of the brain. This disease typically occurs suddenly and progresses rapidly, with a survival rate of less than 5%. PAM usually affects children and young adults who were previously healthy and have a history of swimming in heated pools, man-made lakes, or contact with water or mud. However, there have been cases where the disease occurred without any contact with water.^9^ ^,^ ^53^ ^,^ ^58^ *N. fowleri,* a thermophilic FLA, demonstrates a preference for warm environments and is ubiquitously present in soil, water, and air. It has been found on every continent except Antarctica. This amoeba is commonly found in lakes, hot springs, heated pools, as well as in industrial waste and areas rich in organic matter. *Naegleria* spp. have also been found in moist areas in hospital physiotherapy departments, dust samples in hospitals in Brazil, and dental treatment units. The amoeba can tolerate temperatures of up to 46ºC and exists in three distinct forms: trophozoite, cyst, and a flagellated form. The flagellated form, which is elliptical and has two flagella, is a transient phase responsible for the spread of the microorganism due to its high mobility. When trophozoites come into contact with water, they undergo a process called exflagellation, transforming into the flagellated form.^9^ ^,^ ^11^ ^,^ ^54^

Despite a few reports, other species are associated with the disease.^9^ *Sappinia pedata* and *Vahlkampfiid amoeba* have been confirmed as newly recognised human pathogens. *S. pedata* can cause GAE, but only one case has been reported, with the patient surviving without long-term consequences.^57^ The *Vahlkampfiid amoeba*, classified as *Tetramitus ovis* by DNA sequencing, has been isolated from contact lens cases.^73^ *Paravahlkampfia* sp. was isolated from intestinal tracts of lizards and corneal keratitis samples of patients without contact lenses.^66^ Additionally, *P. francinae* was isolated from the cerebrospinal fluid (CSF) of patients suspected of having viral meningitis. Cell culture revealed an amoeba with morphology similar to that of the *Vahlkampfid* group, and *P. francinae* was identified by DNA sequencing.^55^

FLAs, the genus *Acanthamoeba* has been the subject of extensive research, providing consistent data on its distribution, biology, pathogenesis, and ecological impact on the environment.^9^ ^,^ ^53^ ^,^ ^73^ ^,^ ^74^ ^,^ ^75^

*Acanthamoeba* spp. are ubiquitous protists widely distributed in diverse environments, including fresh water, ocean sediment, dust, moist soil, air, invertebrates and invertebrate microfauna.^8^ ^,^ ^9^ ^,^ ^11^ ^,^ ^54^ Their life cycle consists of two stages, a vegetative trophozoite stage, in which the organism feeds and multiplies, and a metabolically inactive cyst stage, which enables the amoeba not only to survive without nutrients and withstand desiccation and heat, but also to resist disinfection and treatment.^76^ Generally, *Acanthamoeba* spp. feed on bacteria, algae and fungi under aerobic and anaerobic conditions and under extreme conditions concerning pH, salinity and temperature. The trophozoites have a structure similar to professional phagocyte cells. They contain granuloplasm with cell organelles and hyaloplasm that produce pseudopods. The nucleus has a prominent, dense central nucleolus, with finely granular chromatin and two Golgi complexes on opposite sides of the nucleus in one plane. The mitochondria are oval or round-shaped with typically tubular cristae and intra-crystal inclusions. Additionally, there are smooth and rough endoplasmic reticula, free ribosomes, digestive vacuoles, lysosomes, and microtubules. The cytoplasm contains lipid droplets, polysaccharide reserves, and a prominent contractile vacuole that functions in the osmoregulation of the cell.^77^ ^,^ ^78^ ^,^ ^79^ A characteristic feature of this genus is the presence of spiny surface projections called acanthopodia, which are built up by actin fibres and networks.^80^ They play a crucial role in the infection process when interacting with the host cell surfaces. However, when environmental conditions become unfavourable, the trophozoite transforms into highly resistant cysts. These cysts have a double-layered wall and an outer ectocyst, allowing them to maintain viability in the natural environment for at least 25 years.^81^

The genus *Acanthamoeba* currently consists of around 30 species and genotypes (T1 to T23), which are identified based on 18S rDNA sequencing.^82^ Some of these species and genotypes have the potential to cause diseases, leading to severe and often fatal conditions such as GAE and pneumonia, renal infections, skin injuries, and sinusitis in immunosuppressed individuals. The T4 genotype appears to be the most prevalent in various environments and is the most common genotype found in human infections.^82^ *Acanthamoeba* keratitis amoebic occurs in immunocompromised individuals. The greatest populations at risk are soft contact lens wearers, who have been mainly described in industrialised countries. At the onset of the infection, the clinical diagnosis is difficult to assess due to the lack of distinct pathological signs, mainly due to the similarity with herpetic keratitis and with bacterial or fungal infections, leading to insufficient treatment and worsening of clinical conditions. The prognosis is usually unfavourable when clinical suspicion is late and may result in vision loss.^67^ ^,^ ^69^ ^,^ ^83^ *Acanthamoeba* infections outside of the cornea have been rarely described. GAE occurs in patients with immune suppression and chronic diseases such as HIV infection, haematological neoplasms, organ transplantation, use of steroids or other immunosuppressive therapy, systemic lupus erythematosus, diabetes mellitus, prolonged and excessive use of antibiotics, chronic alcoholism, liver cirrhosis, malnutrition, pregnancy, surgical trauma, burns, wounds, or radiotherapy.^9^ ^,^ ^11^ ^,^ ^58^ ^,^ ^70^ ^,^ ^76^

Freshwater amoebae as host for other microorganisms

Freshwater amoebae typically inhabit biofilms, where they consume a variety of microorganisms, including bacteria, algae, nematodes, fungi, cyanobacteria, rotifers ciliates, and other protozoa.^74^ ^,^ ^75^ They capture their prey through phagocytosis, ingesting them and transferring them to lysosomal compartments for digestion. However, some of these microorganisms have evolved mechanisms to evade the microbicidal activity of amoebae, similar to those used by pathogens for survival within macrophages. Recent studies have described the implications of these interactions between FLA and their endosymbiontss on public and environmental health.^75^ ^,^ ^84^ A variety of FLAs are known to act as vehicles for endosymbionts. For instance, *B. mandrillaris* can serve as a host for the pathogenic *Legionella pneumophila* and support the intracellular growth of a *Chlamydia*-like bacterium, *Simkania negevensis*.^85^ ^,^ ^86^ Similarly, *N. fowleri* can provide an intracellular environment conducive to the multiplication of *L. pneumophila*. Notably, the association of *N. fowleri* with *L. pneumophila* has shown no increase in pathogenic potential in mice following intranasal inoculation nor an enhancement of bacterial virulence.^87^ Additionally, *Protochlamydia naegleriophila* sp. nov., recognised as an etiological agent for pneumonia, has been isolated from *Naegleria lovaniensis*,^88^ while *V. cholerae* can survive in *Naegleria* sp. amoeba, after encystation.^89^ Moreover, *V. vermiformis* interacts with fungi such as *Aspergillus fumigatus*, *Candida* spp., and *Fusarium oxysporum*, as well as with bacteria such as *Campylobacter jejuni* and *Mycobacterium leprae*, which can remain viable and virulent within *V. vermiformis* cysts.^56^ ^,^ ^90^

In recent decades, *Acanthamoeba* spp have garnered increasing attention due to their ability to serve as hosts or reservoirs for a variety of microbes, including viruses (*e.g.*, *mimivirus*, *coxsackieviruses*, *adenoviruses*, *poliovirus*, *echovirus*, *enterovirus,* vesicular stomatitis virus, etc.), bacteria (*e.g.*, *Aeromonas*, *Bacillus*, *Bartonella*, *Burkholderia*, *Coxiella*, *Campylobacter*, *Chlamydophil*a, *E. coli*, *Flavobacterium*, *Helicobacter*, *Legionella*, *Listeria*, *Staphylococcus*, *Mycobacterium*, *Pasteurella*, *Prevotella*, *Porphyromonas*, *Pseudomonas*, *Rickettsia*, *Salmonella*, *Shigella*, *Vibrio*, etc.), protists (*e.g.*, *Cryptosporidium* and *Toxoplasma gondii*), and yeast (*e.g.*, *Cryptococcus*, *Blastomyces*, *Sporothrix*, *Histoplasma*, *Streptomyces*, etc.)._^73^_ ^,^ ^74^ ^,^ ^90^ ^-^ _^91^_,_^92^_,_^93^_,_^94^_,_^95^_,_^96^_ Several pathogens can survive within *A. castellanii* using mechanisms similar to those employed by macrophages. For example, *L. pneumophila* relies on the Dot/Icm type IV secretion system for intracellular proliferation in both human macrophages and *Acanthamoeba* spp.^96^ Additionally, *L. pneumophila* is phagocytosed by *Acanthamoeba* via coiling phagocytosis, but it can hinder phagosome-lysosome fusion.^97^ Similarly, *Vibrio cholerae* can evade amoeboid phagosome killing by neutralising proteolytic enzymes, altering local pH, and counteracting reactive nitrogen and oxygen species that might otherwise eradicate internalised bacteria.^98^ Moreover, the invasion of *A*. *castellanii* or macrophages by *Mycobacterium* spp. shows significant similarities at the transcriptional and post-translational levels.^99^ *A. castellanii* is also capable of engulfing pathogenic fungi, such as *Cryptococcus neoformans*, *Histoplasma capsulatum*, *Blastomyces dermatitides*, *Fusarium solani*, and *Sporothrix schenckii*.^74^ Notably, from the host’s perspective, macrophages and amoebae exhibit comparable interaction patterns, which may have significant implications for understanding infections in mammals.^74^ Recently, it has been reported that *Leishmania* spp. can evade the biochemical/lytic activity of *A. castellanii* and replicate within the amoeba^100^ (Figure).

**Figure:**
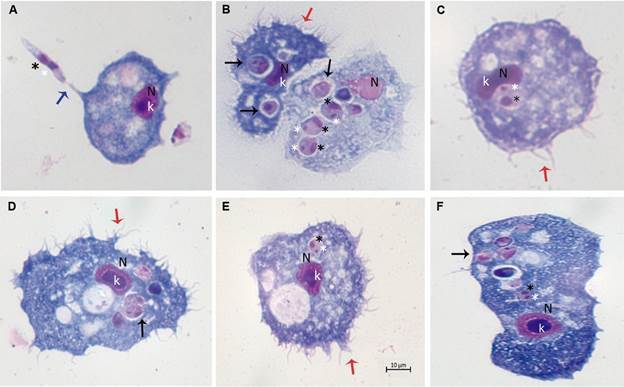
Photomicrographs of *Acanthamoeba castellanii* infected with *Leishmania* spp., parasite ratio of 1:10, stained with Panoptic fast staining. (A) *L. major*; (B, C and F) *L. braziliensis*; (D) *L*. *amazonensis*; (E) *L. infantum*. V: vacuole; N: amoebae nucleus; K: amoeba karyosome; Black star: *Leishmania* nucleus; Write star: *Leishmania* kinetoplast; Black arrowheads: intracellular the rounded/oval body of *Leishmania* inside vacuoles or in the cytosol; Red arrowheads: pine-like pseudopods (acanthopodia); Blue arrowheads: interactions via flagella; Scale bar 10 µm.

These interactions suggest that the innate immune pathway is well conserved and that *Leishmania* spp. may interact within hosts through universal receptors found in professional phagocytes, including *Acanthamoeba* spp. Both *A*. *castellanni* and *A. polyphaga* can engulfed *L. braziliensis*, *L. amazonensis*, *L. infantum*, and *L. major* promastigotes, facilitating their intracellular conversion to an amastigote-like state and supporting their intracellular multiplication via primitive metabolic pathways.^100^ Studies have shown that interactions between pathogens and *Acanthamoeba* can preserve virulence factors and enhance microbial pathogenicity. Consequently, *Acanthamoeba* species may act as universal hosts for certain pathogenic microorganisms, connecting environmental factors to host virulence.^101^ Interestingly, another example of amoebae is *Neoparamoeba* spp. that harbours a kinetoplastid is *Perkinsiella* amoebae-like endosymbionts. These endosymbionts are believed to be related to the kinetoplastid *Ichthyobodo*, a free-living flagellate. These endosymbionts seem to be mutually beneficial for both organisms, as suggests a metabolic and cellular interdependence between the amoeba and the kinetoplastid.^102^

Conclusion remarks and perspectives

This review explored historical aspects of current discoveries on FLA. Despite the relatively low number of infections caused by FLA, it is important to recognise that the reported frequency may be significantly underestimated due to the lack of a diagnostic test with adequate specificity and sensibility. Understanding the pathogenesis and pathophysiology of these infections at the molecular, cellular, and clinical levels is essential. Enhancing our knowledge of these pathogens will help in developing more effective diagnostic, preventive, and therapeutic interventions. From the host’s perspective, macrophages and amoebas exhibit similar interaction patterns. This similarity may have important implications for understanding infections in mammals. By investigating the molecular basis of the interactions between amoebas and various microorganisms - both pathogenic and non-pathogenic - we can gain insights into the receptors, gene expression, and underlying molecular mechanisms involved. These insights could reveal functions that are also present in mammalian cells. Additionally, studying amoebas may provide a complementary perspective on the evolutionary pathways that pathogens have taken to adapt to different hosts.
